# Epigenetic histone acetylation modulating prenatal Poly I:C induced neuroinflammation in the prefrontal cortex of rats: a study in a maternal immune activation model

**DOI:** 10.3389/fncel.2022.1037105

**Published:** 2022-11-28

**Authors:** Yueqing Su, Jiamei Lian, Shiyan Chen, Wenchang Zhang, Chao Deng

**Affiliations:** ^1^Fujian Maternity and Child Health Hospital, College of Clinical Medicine for Obstetrics & Gynaecology and Paediatrics, Fujian Medical University, Fuzhou, China; ^2^Fujian Provincial Key Laboratory of Environment Health, School of Public Health, Fujian Medical University, Fuzhou, China; ^3^Antipsychotic Research Laboratory, Illawarra Health and Medical Research Institute, Wollongong, NSW, Australia; ^4^School of Medical, Indigenous and Health Sciences, and Molecular Horizons, University of Wollongong, Wollongong, NSW, Australia; ^5^Department of Neurology, The First Affiliated Hospital of Fujian Medical University, Fuzhou, China

**Keywords:** histone acetylation, prenatal Poly I:C exposure, neuroinflammation, NF-κB, NLPR3

## Abstract

**Introduction:** Neuroinflammation in the central nervous system, particularly the prefrontal cortex (PFC), plays a role in the pathogenesis of schizophrenia, which has been found to be associated with maternal immune activation (MIA). Recent evidence suggests that epigenetic regulation involves in the MIA-induced neurodevelopmental disturbance. However, it is not well-understood how epigenetic modulation is involved in the neuroinflammation and pathogenesis of schizophrenia.

**Methods:** This study explored the modulation of histone acetylation in both neuroinflammation and neurotransmission using an MIA rat model induced by prenatal polyriboinosinic-polyribocytidylic acid (Poly I:C) exposure, specifically examining those genes that were previously observed to be impacted by the exposure, including a subunit of nuclear factor kappa-B (*Rela*), Nod-Like-Receptor family Pyrin domain containing 3 (*Nlrp3*), NMDA receptor subunit 2A (*Grin2a*), 5-HT2A (*Htr2a*), and GABAA subunit β3 (*Gabrb3*).

**Results:** Our results revealed global changes of histone acetylation on H3 (H3ace) and H4 (H4ace) in the PFC of offspring rats with prenatal Poly I:C exposure. In addition, it revealed enhancement of both H3ace and H4ace binding on the promoter region of *Rela*, as well as positive correlations between *Rela* and genes encoding histone acetyltransferases (HATs) including CREB-binding protein (CBP) and E1A-associated protein p300 (EP300). Although there was no change in H3ace or H4ace enrichment on the promoter region of *Nlrp3*, a significant enhancement of histone deacetylase 6 (HDAC6) binding on the promoter region of *Nlrp3* and a positive correlation between *Nlrp3* and *Hdac6* were also observed. However, prenatal Poly I:C treatment did not lead to any specific changes of H3ace and H4ace on the promoter region of the target genes encoding neurotransmitter receptors in this study.

**Discussion:** These findings demonstrated that epigenetic modulation contributes to NF-κB/NLRP3 mediated neuroinflammation induced by prenatal Poly I:C exposure *via* enhancement of histone acetylation of H3ace and H4ace on *Rela* and HDAC6-mediated NLRP3 transcriptional activation. This may further lead to deficits in neurotransmissions and schizophrenia-like behaviors observed in offspring.

## Introduction

Accumulated epidemiological evidence has suggested that maternal immune activation (MIA) induced by pregnancy infection and other inflammatory processes is a particular environmental pathogenic factor of neuropsychiatric disorders, including schizophrenia and autism (Estes and McAllister, 2016). The epigenetic processes in the placenta have been reported to be closely associated with infant neurodevelopment (Lester and Marsit, 2018), while maternal immune activation could cause epigenetic differences in rodent brains (Basil et al., 2014; Werner et al., 2017). Thus, epigenetic modulation could be one of the potential mechanisms underlying environmentally induced diseases, including MIA-induced neuropsychiatric disorders (Khoury and Galea, 2016; Cavalli and Heard, 2019); however, the underlying epigenetic mechanism is still not fully understood (Thapar et al., 2017).

Histone acetylation is a well-known process for chromatin remodeling and is important in the epigenetic regulation of post-translational modification associated with chromatin transcriptional activity and gene expression (Brehm et al., 1998; Su et al., 2016). Epigenetic histone acetylation on H3 (H3ace) or H4 (H4ace) plays an important role in long-term memory formation, cognitive ability, and synaptic plasticity (Levenson et al., 2004; Bredy et al., 2007; Green et al., 2017; Aggarwal et al., 2019). A study using the postmortem PFC samples from schizophrenia patients has shown a significant hypoacetylation of H3K9K14 that is correlated with the expression of several schizophrenia-related genes, such as *GAD1* (glutamic acid decarboxylase 1) and *HTR2C* (5-hydroxytryptamine receptor 2C; Tang et al., 2011). On the other hand, prenatal Poly I:C exposure has been reported to cause elevation of histone H3K9K14 acetylation at the promoter regions of several schizophrenia-related genes, such as *Disc1*, *Nr2f1*, *Gria1*, and *Gria2* in the hippocampus of juvenile (24 days old) offspring before the onset of behavioral deficits, but without changes in acetylation of histone H4K8 at these promoter loci (Tang et al., 2013). Histone acetylation is also involved in the regulation of innate and adaptive immune pathways (Shakespear et al., 2011), particularly the activation of pro-inflammatory gene expression mediated by toll-like receptors (TLRs; Bode et al., 2007). Similar to other histone modifications, epigenetic histone acetylation is dynamic, regulated by acetylating or deacetylating lysine residue on the N terminal of histones, which is carried out by type A histone acetyl transferases (HATs) or histone deacetylases (HDACs) in the nucleus, respectively (Roth et al., 2001; Shen et al., 2015). Nineteen type A HATs, which are sub-classified into five major families, have been identified in mammals (Roth et al., 2001; Sheikh, 2014). Among these, CREB-binding protein (CBP), E1A-associated protein p300 (EP300), General Control Non-depressible 5 (GCN5), Tip60, and P300/CBP-associated factor (PCAF) have been described as playing a crucial role in neurodevelopment and been found to have a relatively high expression in the brain of rats (Schneider et al., 2013; Sheikh, 2014). It has been reported that CBP and EP300, the two closely related HATs, are directly involved in the pathologies of neuropsychiatric disorders (Valor et al., 2013). Tip60 played a critical role in controlling specific gene expression profiles, which were essential for functions of the nervous system (Lorbeck et al., 2011). GCN5 participated in the regulation of transcriptional programs in neural stem cells (Martínez-Cerdeño et al., 2012), and the loss of GCN5 contributed to the acceleration of neuronal apoptosis (Wu et al., 2017).

As a group of enzymes that decrease the acetylation status of proteins, there are 18 HDACs which are categorized as four classes of HDACs: the class I Rpd3-like proteins (HDAC1–3 and HDAC8), the class II Hda1-like proteins (HDAC4–7, HDAC9, and HDAC10), the class III Sir2-like proteins (SIRT1–7), and the class IV protein (HDAC11; Seto and Yoshida, 2014). Emerging evidence has also suggested that several HDACs, in particular HDAC2, HDAC4, and HDAC6, regulated transcriptional expression of genes linking to neuronal function and neuroinflammation through mediated the acetyl group in the histone, which were associated with holistic neuronal function and the molecular pathology of neuropsychiatric disorders (Dai et al., 2021; Gupta et al., 2021; Kumar et al., 2022). For instance, HDAC2 was reported to alter hippocampal neuronal transcription and microglial activity in neuroinflammation-induced cognitive dysfunction, while inhibition of HDAC2 activation could alleviate neuroinflammatory responses in lipopolysaccharide (LPS) stimulated microglia (Jiao et al., 2018; Sun et al., 2019). HDAC4 represses genes encoding constituents of central synapses that are essential for synaptic plasticity and memory (Sando et al., 2012). Persistent upregulation of HDAC4 was associated with the release of pro-inflammatory factors interleukin (IL)-1β, IL-6, interferon γ (IFN-γ), and tumor necrosis factor α (TNF-α) in the brain of adult zebrafish after stress exposure (Yang et al., 2020). In addition, HDAC6 also contributed to the transcriptional regulation of inflammatory genes (Youn et al., 2015; Wang et al., 2021). Inhibition of HDAC6 by its selective inhibitor Tubastatin A alleviated LPS-induced brain inflammation in mice (Song et al., 2019). Interestingly, other than modulating the acetyl group in the histone, HDAC2 and HDAC6 could also be directly recruited to the specific promoter region and act as a cofactor in a complex to regulate the expression of target genes (Palijan et al., 2009; Chen et al., 2013; Ecker et al., 2021).

Neuroinflammation in the central nervous system, particularly the prefrontal cortex (PFC), is involved in the pathogenesis of schizophrenia, in which the nuclear factor kappa-B (NF-κB) and related pathways play an important role (Murphy et al., 2021; Vallée, 2022). Exposure to polyriboinosinic-polyribocytidylic acid (Poly I:C), a mimic of viral dsRNA, during a specific period of pregnancy is one of the commonly used animal models for studying the pathogenesis of MIA-induced neuropsychiatric disorders, including schizophrenia and autism (Reisinger et al., 2015). Since Poly I:C could be specially recognized by the transmembrane protein TLR3, it thus induces the activation of NF-κB and modulated IL-1β mRNA expression and pro-IL-1β synthesis (Akira and Takeda, 2004; Su et al., 2022). The activation of NF-κB after ligation of the TLR3 by Poly I:C also promoted the production of a Nod-Like-Receptor family Pyrin domain containing 3 (NLRP3), another key inflammasome component in the innate immune system, and lead to the cleavage and release of pro-inflammatory cytokines such as pro-IL-1β (Rajan et al., 2010). Recent studies suggested that activation of the NF-κB/NLRP3 pathway plays a key role in Poly I:C induced long-term elevation of neuroinflammation in a number of brain regions including the PFC (Giridharan et al., 2020; Su et al., 2022; Szabó et al., 2022). Meanwhile, there is evidence that the MIA-elicited neuroinflammation may induce deficits in serotonergic, glutamatergic, and gamma-aminobutyric acid (GABA)-ergic neurotransmission, which could be the pathophysiological changes underlying behavioral abnormalities caused by prenatal Poly I:C challenge (Hao et al., 2019; Nakagawa et al., 2020; Ravaccia and Ghafourian, 2020; Su et al., 2022). In line with the activation of the NF-κB/NLRP3 neuroinflammation pathway could be modulated by histone acetylation (Chen et al., 2018; Fang et al., 2021), histone acetylation is also involved in mediating neurotransmitter receptors such as serotonin 5-HT_1A_ (5-HT_1A_R), N-methyl-D-aspartate (NMDAR) and GABA_A_ (GABA_A_R) receptors that play a key role in the pathophysiology of mental illness (Tsuji et al., 2014; Imamura et al., 2016; Auta et al., 2018). Prenatal Poly I:C (5.0 mg/kg) exposure at gestational day (GD) 17 increased global HDAC activity, particularly in female adult offspring, but it is unclear which specific HDACs were mediating this effect (Pujol Lopez et al., 2016). To date, no studies have explored the modification role of histone acetylation in the activation of neuroinflammation and deficiency of neurotransmission induced by prenatal Poly I:C exposure. Previous studies have revealed sex differences in behaviors and neuropathological changes of rodent MIA models for schizophrenia (Kokras and Dalla, 2014; Gogos et al., 2020). However, to avoid the potential impacts of estrogens, the majority of studies have been performed in male offspring only but with limited research in females (Prendergast et al., 2014). In our recent study, along with a list of behavioral deficits, activation of the NF-κB/NLRP3 pathway and abnormal expression of neurotransmission receptors were observed in the PFC of female juvenile rats with prenatal Poly I:C exposure (Su et al., 2022). Therefore, to investigate the role of modulation of histone acetylation on neuroinflammation and neurotransmission, this study evaluated the effects of prenatal Poly I:C exposure on the global level of H3ace and H4ace, expression of HATs and HDACs, and the enrichment of H3ace, H4ace, and HATs/HDACs on the promoter region of specific genes linking to the NF-κB/NLRP3 pathway and neurotransmitter receptors in the PFC of female offspring.

## Materials and Methods

### Animals and treatment

The methods for establishing a Poly I:C rat model were reported previously, which showed a phenotype with schizophrenia-like behavioral deficits in both adolescent and adult offspring (Osborne et al., 2019a, b; Lian et al., 2022; Su et al., 2022). In brief, pregnant Sprague-Dawley rats at GD8 were purchased from the Animal Resource Centre (Perth, Australia). At GD15 (mid-late gestation), these pregnant rats were randomly assigned into two groups: (1) six animals received an intraperitoneal (IP) injection with 5 mg/kg Poly I:C (Invivogen, Toulouse, France), dissolved in 0.2 ml 1% phosphate buffer saline (PBS); and (2) the other six were injected with an equivalent volume of PBS. After weaning on postnatal day (PD) 21, female offspring pups were housed in Techniplast GR1800 double-decker rat individually ventilated cages (IVCs) under environmentally controlled conditions (22°C, light cycle from 07:00 h to 19:00 h and dark cycle from 19:00 h to 07:00 h). Female offspring from different dams were randomly selected in this study (saline *n* = 6, Poly I:C *n* = 6). Each cage housed two rats from the same treatment group, and a divider separated the cage into two chambers of equal size, each with its own food hopper and water bottle for *ad libitum* access to water and standard laboratory chow diet. The two rats could see, hear, and smell each other through perforated holes in the divider.

### Brain tissue collection

Rats were euthanized by isoflurane anesthesia and underwent decapitation on PD60. The brain was immediately frozen in liquid nitrogen and then stored at −80°C. Brain tissue was dissected at −10.5°C ± 1.5°C into 500 nm coronal sections in a cryostat (Leica Biosystems, Nussloch, Germany). According to the rat brain atlas (Paxinos and Watson, 2007), the PFC from each animal was collected and stored in a −80°C freezer until use.

### Western blot

Global changes of H3ace and H4ace were analyzed by Western Blot. In brief, brain tissues were homogenized using a Precelly 24 homogenizer (Bertin Technologies, Montigny-le-Bretonneux, France) having added 400 μl lysis buffer, which was made up of 0.3 M phenyl-methylsulfonyl fluoride (33.3 μl, Sigma–Aldrich, St. Louis, USA), 50 mM β-Glycerophosphate (100 μl, Invitrogen, Camarillo, USA), NP40 cell lysis buffer (9.8 ml, Invitrogen, Camarillo, USA), and protease inhibitor cocktail (100 μl, Sigma–Aldrich, St. Louis, USA). An 8%–12% sodium dodecyl sulfate-polyacrylamide gel (Bio-Rad, Hercules, USA) was applied for protein electrophoresis. After being trans-blotted onto a polyvinylidene difluoride membrane (Bio-Rad, Hercules, USA) and blocked with 5% skim milk in Tris-buffered saline with 0.1% Tween-20, the separated protein was incubated overnight with anti-H3ace (#MAB06-599, 1:1,000; Millipore, Billerica, USA), anti-H4ace (#MAB06-598, 1:1,000; Millipore, Billerica, USA) or mouse anti-actin polyclonal antibodies (#MAB1501, 1:10,000; Millipore, Billerica, USA). The separated protein was then incubated with horseradish peroxidase (HRP)-labeled anti-rabbit antibody (# AB97051, 1:5,000; Abcam, Cambridge, UK) for 1-h the next day. Western blot results were visualized using an Amersham GelImager 600 (GE Healthcare, Pittsburgh, USA), quantified by a Fiji Image processing package, and normalized by both β-Actin and the control group.

### Chromatin immunoprecipitation (ChIP)-quantitative real-time PCR (qPCR)

ChIP-qPCR was used to evaluate the enrichment of H3ace, H4ace, or HDAC6 binding on the promoter region of target genes of both the NF-kB/NLRP3 pathway and neurotransmitter receptors. These included *Rela*, *Nlrp3*, NMDA receptor subunit 2A (*Grin2a*), 5-HT2A (*Htr2a*), and GABAA subunit β3 (*Gabrb3*), because their mRNA level in the PFC had been previously observed to be significantly impacted by prenatal Poly I:C exposure in the PFC of female juvenile offspring rats (Su et al., 2022). In accordance with the manufacturer’s instructions for the EpiQuik Tissue ChIP Kit, EpiQuik Tissue Acetyl-Histone H4 ChIP Kit, and EpiQuik Tissue Acetyl-Histone H3 ChIP Kit (Epigentek, New York, USA) which were specific for tissue samples, 20 mg of brain tissue was cross-linked by 1,000 μl 1% formaldehyde (10 ml 1,640 cell culture medium +270 μl 37% formaldehyde) and disaggregated using a Precellys^®^24 homogenizer (Bertin Technologies, Montigny-le-Bretonneux, France). Cell lysis was performed and sheared to a 200–1,000 bp fragment by a Branson 450 Digital Sonifier (Emerson, St. Louis, USA). The cross-linked DNA was then transferred to a strip well which had been binding with chip-grade Anti-Acetyl Histone H4, Anti-Acetyl Histone H3, HDAC6 (ab239362, Abcam, Cambridge, UK), or anti-mouse IgG (negative control) for protein/DNA immunoprecipitation. The fragmented DNA binding with these specific antibodies was then reversed and purified for further analysis. qPCR was performed in duplicate for each sample with SYBR Green Master Mix (Qiagen, Germantown, USA) to determine the target gene that was bound with H3ace, H4ace, or HDAC6 on its promoter region [within upstream 2,000 kb of transcription start site (TSS)]. The primers were designed on the relatively higher peak regions of the histone markers in the database of ChIP-Atlas^1^ through the Primer-BLAST in the National Center for Biotechnology Information (NCBI) and synthesized by Sigma-Aldrich (St. Louis, USA; Supplementary Figure 1).

The ChIP-qPCR pre-experiments were conducted in three samples randomly taken from the control group, which revealed a relatively strong binding of: (1) both H3ace and H4ace on the promoter region of −1,097 to −1,007 bp in *Nlrp3*, and −765 to −651 bp in *Rela* (Supplementary Figures 2A,B); (2) HDAC6 on the promoter region of −1,097 to −1,007 bp in *Nlrp3* (Supplementary Figure 2C); (3) of H3ace on the promoter region of −1,318 to −11,168 bp in *Grin2a*, and −1,380 to −1,274 bp in *Htr2a* (Supplementary Figure 3A); and (4) of H4ace on the promoter region of −1,372 to −1,279 bp in *Gabrb3* (Supplementary Figure 3B). Therefore, the enrichment differences in these regions between all samples in the two groups were analyzed in the following experiments. ChIP-qPCR data was presented as a percentage of input: % Input = 2^∧^ (−ΔCt [normalized ChIP]) ΔCt [normalized ChIP] = (Ct [ChIP] − (Ct [Input] − Log2^(Input Dilution Factor)^)).

### Reverse transcription-qPCR (RT-qPCR)

RT-qPCR was applied for measuring the mRNA level of genes encoding HATs and HDACs, including *Gcn5*, *Pcaf*, *P300* (EP300), *Crebbp* (CBP), *Tip60*, *Hdac2*, *Hdac4*, and *Hdac6*. In brief, total RNA was extracted using the PureLink^TM^ RNA Mini Kit (#12183025; Invitrogen, Camarillo, USA), and quantified by NanoDrop 2000 (Thermo Fisher Scientific, Waltham, USA). It was then converted to cDNA using a High-Capacity cDNA Reverse transcription kit (# 4368814; Thermo Fisher Scientific, Waltham, USA). qRT-PCR was run on a Quant Studio^TM^ qRT-PCR system (Thermo Fisher Scientific, Waltham, USA) with the following conditions: 95°C 10 min, 40 cycles of 95°C 15 s, and 60°C 1 min. All samples were measured in duplicate using SYBR^TM^ Green PCR Master Mix (Thermo Fisher Scientific, Waltham, USA) for *p300* (forward 5’-ACCTTCTCCTGTCCTAGCCGTAC-3’, reverse 5’-AATTGCTGTTGCTGCTGGTTGTTG-3’), *Tip60* (forward5’-GCCAAGACACCTACCA AGAACGG-3’, reverse5’-GGTTGAAGCGGAGTGTGTCTGG-3’), *Crebbp* (forward5’-CATTGGAAGCCTCAGCACGATACC-3’, reverse 5’-GGGTCAGGAGTGGGAAGATGG-3’), *Pcaf* (forward 5’-AACGTGCCGCAGTTCTGGAC-3’, reverse 5’-GATGGTGAAGACGGAGCGAAGC-3’), and *Gcn5* (forward5’-TCCTGCTGCCACCATCCTCTATG-3’, reverse 5’-CTTGACCTTAGCCATCAGCACCAG-3’). TaqMan^®^ Gene Expression Assays (Thermo Fisher Scientific, Waltham, USA) were used for *Hdac2* (Rn01407870_g1), *Hdac4* (Rn01427040_m1), *Hdac6* (Rn01528284_g1), *β*-actin (Hs01060665_g1), and *Gapdh* (Rn01775763_g1). The relative mRNA expression of each target gene was normalized by both *β*-actin and *Gapdh*, then calculated according to the 2^−ΔΔCT^ method.

### Statistical analysis

Data were analyzed and graphed using GraphPad Prism 7.04 (GraphPad Software Inc., San Diego, USA). The outliers were identified and removed using Boxplot on SPSS 25.0 (IBM, New York, USA). Data distribution was examined by a Kolmogorov-Smirnov test. For all data with normal distribution, a student *t*-test was used to compare the difference between Poly I:C and saline-treated groups, and a Pearson correlation was used to determine the relationships between the various measurements. For those without normal distribution, a nonparametric Mann–Whitney *U* test and Spearman correlation were performed. Statistical significance was accepted when *p* < 0.05. The results were presented as the mean ± SEM.

## Results

### Global level of H3ace and H4ace

**Figure 1:**
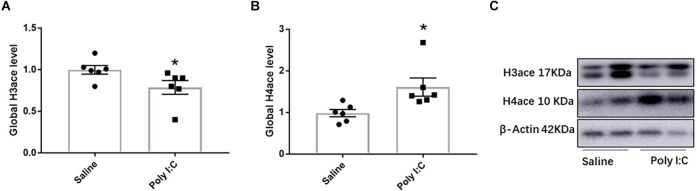
Effect of prenatal Poly I:C exposure on the global level of H3ace and H4ace in the prefrontal cortex of female juvenile rats. **(A)** H3ace, **(B)** H4ace, and **(C)** representative Western-blotting images. Data were presented as mean ± SEM (*n* = 6/group). The molecular weights of H3ace, H4ace, and Actin are 17 kDa, 10 kDa, and 43 kDa, respectively. *p < 0.05, Poly I:C vs. Control.

Compared with the control, the global level of H3ace was significantly decreased in the Poly I:C group (*t* = 2.162, df = 10, *p* = 0.028; Figures 1A,C; Supplementary Figure 4). However, rats with prenatal Poly I:C exposure exhibited a significant increase in the global level of H4ace (*t* = 2.654, df = 10, *p* = 0.024; Figures 1B,C; Supplementary Figure 4). These results showed the global changes of H3ace and H4ace in the opposite directions, which indicated that prenatal Poly I:C treatment-induced changes in the epigenetic regulation of histone acetylation in the female adolescent offspring. Therefore, ChIP-qPCR experiments were performed to investigate the role of H3 or H4 histone acetylation on the expression of targeted genes caused by prenatal Poly I:C exposure.

### Histone acetylation on the promoter region of target genes linking to the NF-κb/NLRP3 pathway

**Figure 2:**
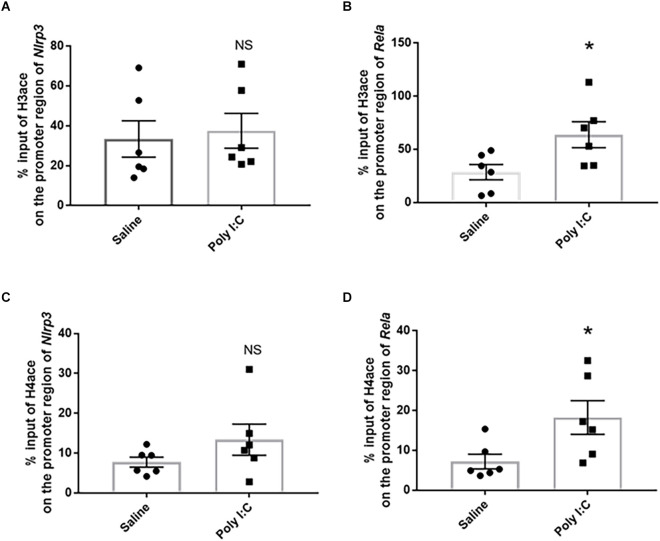
Histone acetylation on the promoter region of the target genes associated with the NF-kB/NLPR3 pathway. Enrichment of acetylated histone 3 on **(A)** *Nlrp3* and **(B)** *Rela*, as well as enrichment of acetylated histone 4 on **(C)** *Nlrp3* and **(D)** *Rela*. Data were presented as mean ± SEM (*n* = 6/group). NS, no significance; *p < 0.05, Poly I:C vs. Control.

The % input of H3ace on the region of *Nlrp3* promoter was 37.490 ± 8.836% in the rats with prenatal Poly I:C exposure, compared with 33.390 ± 9.121% in the control, while no significant difference was observed between the two groups (*t* = 0.325, df = 10, *p* = 0.752; Figure 2A). However, a significant increase of H3ace binding on the promoter region of *Rela* (−765 to −651) was observed between the Poly I:C (63.730 ± 12.180%) and saline groups (28.540 ± 7.283%; *t* = 2.479, df = 10, *p* = 0.036; Figure 2B).

Similar to H3ace, the average H4ace % input value on the promoter region of *Nlrp3* was also not significantly different between the two groups (Poly I:C 13.380 ± 3.893% vs. Saline 7.760 ± 1.261%; *t* = 1.374, df = 10, *p* = 0.199; Figure 2C); while prenatal Poly I:C exposure significantly increased the enrichment of H4ace binding on the promoter region of *Rela* (Poly I:C 18.250% ± 4.231% vs. Saline 7.203% ± 1.843%; *t* = 2.394, df = 10, *p* = 0.036; Figure 2D). In consideration of the upregulated mRNA expression of *Nlrp3* and *Rela* found in the previous study (Su et al., 2022), these data provided evidence that histone acetylation is involved in neuroinflammation activated by prenatal Poly I:C exposure through enriching both H3ace and H4ace on the promoter region of the *Rela* gene.

### Histone acetylation on the promoter region of target genes encoding neurotransmitter receptors

**Figure 3:**
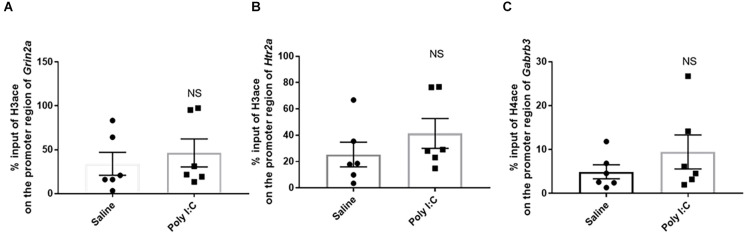
Histone acetylation on the promoter region of the genes encoding neurotransmitter receptors. Enrichment of acetylated histone 3 on **(A)** the NMDA receptor subunit *Grin2a* and **(B)** 5-HT_2A_ receptor *Htr2a*, as well as enrichment of acetylated histone 4 on **(C)** the GABAA receptor subunits *Gabrb3*. Data were presented as mean ± SEM (*n* = 6/group). NS, no significance; Poly I:C vs. Control.

There was no significant difference in the % input of H3ace binding on the region −1,318 to −11,168 in *Grin2a* (46.390% ± 15.980%) in the rats with prenatal Poly I:C exposure compared with the control (33.980% ± 13.060%; *t* = 0.601, df = 10, *p* = 0.580; Figure 3A). Similarly, the % input of H3ace binding on the region of −1,380 to −1,274 in *Htr2a* was not significant between the two groups (41.370% ± 11.330% in Poly I:C rats vs. 25.340% ± 9.382% in the control; *t* = 1.090, df = 10, *p* = 0.602; Figure 3B). For H4ace, there was also no significant difference in the average of % input on the promoter region of *Gabrb3* (9.472% ± 1.594% in Poly I:C vs. 4.943% ± 1.594% in the control; *t* = 1.081, df = 10; *p* = 0.601; Figure 3C).

### The effect on the mRNA expression of genes encoding HATs and HDACs

**Figure 4:**
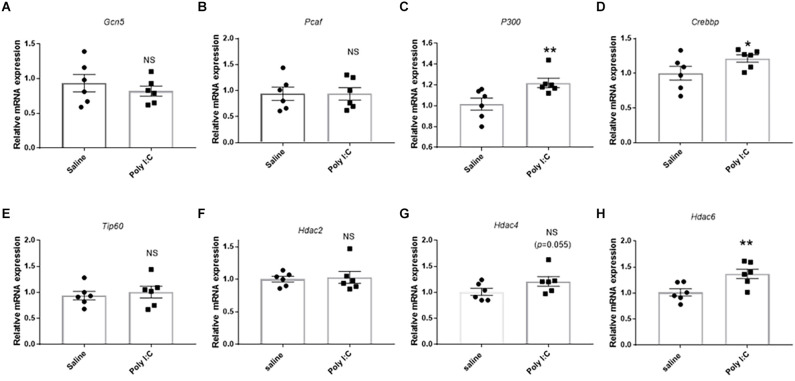
Effect of prenatal Poly I:C exposure on the mRNA expression of histone acetyltransferases (HATs) and deacetylases (HDACs). **(A)** *Gcn5*, **(B)** *Pcaf*, **(C)** *P300*, **(D)** *Crebbp*, **(E)** *Tip60*, **(F)** *Hdac2*, **(G)** *Hdac4*, and **(H)** *Hdac6*. Data were presented as mean ± SEM (*n* = 6 per group). NS, no significance; *p < 0.05, **p < 0.01; Poly I:C vs. Control.

For HATs, no significant difference was observed in the mRNA levels of *Gcn5*, *Pcaf* or *Tip60* in rats treated with Poly I:C treatment compared with the control (all *p* > 0.05; Figures 4A,B,E). However, the mRNA expression of *P300* was significantly higher in the Poly I:C group than the control (*t* = 2.576, df = 10, *p* = 0.014; Figure 4C). Meanwhile, there was also a significant upregulation of *Crebbp* mRNA expression in the rats with prenatal Poly I:C exposure (*t* = 1.894, df = 10, *p* = 0.043; Figure 4D).

For HDACs, although there was no significant difference in the *Hdac2* mRNA expression between the Poly I:C rats and saline rats (*t* = 0.278, df = 10, *p* = 0.393; Figure 4F), an increase of *Hdac4* (*t* = 1.746, df = 10, *p* = 0.055; Figure 4G), and a significant upregulation of *Hdac6* mRNA expression (*t* = 3.056, df = 10, *p* = 0.006; Figure 4H) were observed in the rats with prenatal Poly I:C exposure compared with the control.

### Enrichment of HDAC6 on the promoter region of target genes

**Figure 5:**
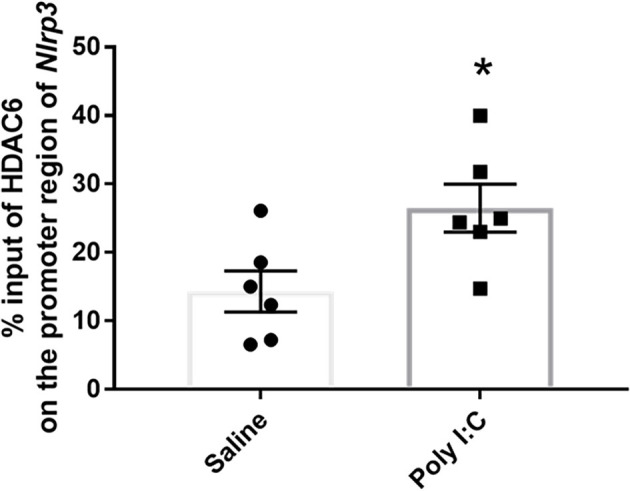
Enrichment of Hdac6 on the promoter region of *Nlrp3*. Data were presented as mean ± SEM (*n* = 6/group). *p < 0.05, Poly I:C vs. Control.

Previously accumulated evidence also implicated the regulation role of HDAC6 on the processes of both inflammation and neurotransmission (Ran and Zhou, 2019; LoPresti, 2020). HDAC6 could act as a cofactor to be recruited to the promoters and activate the target gene expression directly (Palijan et al., 2009; Chen et al., 2013). Further ChIP-qPCR using anti-HDAC6 was performed to investigate the role of upregulated HDAC6 on the dysregulation of neurotransmitter receptors and activation of the NF-kB/NLRP3 pathway induced by prenatal Poly I:C exposure. Compared to the control, there was a significant increase of HDAC6 binding on the promoter region of *Nlrp3* in the rats with prenatal Poly I:C exposure (28.480 ± 3.498 vs. 14.310 ± 3.010; *t* = 2.638, df = 10, *p* = 0.024; Figure 5).

### Correlation analysis

**Figure 6:**
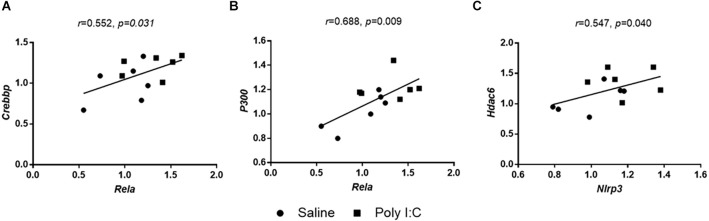
Correlations between the mRNA expression between **(A)** *Nlrp3* and *Hdac6*, **(B)** *Rela* and *p300*, and **(C)** *Rela* and *Crebbp*.

There were positive correlations between *Rela* and *Crebbp* (*r* = 0.552, *p* = 0.031; Figure 6A), as well as *Rela* and *P300* (*r* = 0.688, *p* = 0.009; Figure 6B). These data suggested the increasing enrichment of histone acetylation on the promoter region of *Rela* induced by Poly I:C may be recruited by the HATs of CBP and EP300. Meanwhile, a significant positive correlation of mRNA levels between *Nlrp3* and *Hdac6* (*r* = 0.547, *p* = 0.045; Figure 6C) was observed, suggesting a role of HDAC6 in NLRP3 transcriptional translation since previous evidence showed the epigenetic modification of HDAC6 in neuroinflammation (Dai et al., 2021). However, there were no significant correlations between the mRNA expression of any neurotransmitter receptors and HATs or HDACs (all *p* > 0.05).

## Discussion

It has been evidenced that neuroinflammation mediated by the NF-κB and related pathways plays a role in the pathogenesis of schizophrenia (Murphy et al., 2021; Vallée, 2022). Using an MIA model, this study provided the first evidence that epigenetic histone acetylation of H3 and H4 (H3ace and H4ace, respectively) peaking on the promoter region of *Rela* was involved in the activation of the NF-κB mediated inflammatory pathway in the PFC induced by prenatal Poly I:C exposure. This histone acetylation event was modulated by the specific histone acetyltransferases EP300 (p300) and CBP (*Crebbp*), both of which were type A HATs that originated in the nucleus and showed a typical function of catalyzing the process of transcription-related histone acetylation (Roth et al., 2001). Although there was no specific change in either H3ace or H4ace binding on the promoter region of *Nlrp3* in Poly I:C rats, a significant enhancement of HDAC6 peaking on the promoter region of *Nlrp3* and a significant positive correlation between the higher expression of *Nlrp3* and *Hdac6* were observed. Since HDAC6 could act as a cofactor to be recruited to the promoters and activate the target gene expression directly (Palijan et al., 2009; Chen et al., 2013), these results suggest that prenatal Poly I: C-induced enhancement of neuroinflammation is also associated with HDAC6-mediated NLRP3 transcriptional activation. On the other hand, although prenatal Poly I:C exposure induced abnormal expressions of neurotransmitter receptors *Grin2a, Gabrb3*, and *Htr2a* in the PFC (Su et al., 2022), this study showed no direct H3 and H4 histone acetylation at the promoter regions of genes encoding these neurotransmitter receptors.

The global profile of histone acetylation refers to the acetylation level throughout the transcribed unit of genes and is a ubiquitous hallmark of transcription (Kurdistani and Grunstein, 2003; Kurdistani et al., 2004). It is consistent with previous studies (Tang et al., 2013; Reisinger et al., 2016) that global changes of histone H3 and H4 acetylation in the PFC were observed responding to the prenatal Poly I:C exposure in this study, suggesting the involvement of histone acetylation in the transcriptional changes induced by prenatal Poly I:C exposure (Su et al., 2022). Meanwhile, histone acetylation on different lysine of different core histones is associated with the transcription of biologically related gene groups (Kurdistani et al., 2004). Recent studies also suggested that genome-wide histone acetylation was the consequence of transcription (Martin et al., 2021), therefore a specific state of global histone acetylation may reflect the characteristic changes of gene expression profiles induced by Poly I:C, which may be impacted by the dosages, timing of prenatal exposure, brain regions, or the age of offspring examined. For example, an increased global level of H3ace, but decreased H4ace global levels, have been reported in the hippocampus of adult female mouse offspring with prenatal Poly I:C (20 mg/kg, IP) exposure at GD12.5 (Reisinger et al., 2016). On the other hand, prenatal Poly I:C (5 mg/kg, IP) exposure at GD9 has been reported to lead to a global hypoacetylation of both histones H3 and H4 in the cortex of juvenile but not adult offspring mice, as well as no changes in global levels of H3ace and H4ace in the hippocampus of both juvenile and adult offspring (Tang et al., 2013). Whereas, we observed a decreased global level of H4ace but an increase in H3ace in the PFC of female juvenile rats with prenatal Poly I:C (5 mg/kg, IP) exposure at GD15. Thus, it is necessary to further analyze the effect of prenatal Poly I:C exposure on promoter-located acetylation in individual genes, because the promoter region plays a pivotal role in the regulation of gene expression (MacDonald and Howe, 2009).

Although our previous study has found prenatal Poly I:C (5 mg/kg at GD15) exposure caused abnormal mRNA expression of neurotransmitter receptors *Htr2a*, *Grin2a*, and *Gabrb3* (Su et al., 2022), this prenatal Poly I:C treatment did not lead to any specific changes of H3ace and H4ace on the promoter region of *Htr2a*, *Grin2a*, and *Gabrb3* genes in the PFC of female adolescent rats. It has been reported that a significant increase of acetylated H3 and H4 occurred on the promoter of serotonin transporter in the hippocampus of adult MIA mice (PD84) induced by a single IP injection of 20 mg/kg PolyI:C at GD12.5 (Reisinger et al., 2016). These differences may be explained by the time-dependent character of epigenetic modification, as well as the difference in animal species, age of offspring, brain region examined, or timing and dosages of prenatal Poly I:C challenge (Conti et al., 2014; Richetto et al., 2017).

Toll-like Receptor 3 is the specific pathogen recognition receptor for Poly I:C-induced innate immune response, including leading to activation of the transcription factor NF-κB (Rajan et al., 2010; Topping and Kelly, 2019). NF-κB plays a crucial role in regulating inflammatory pathways, including NLRP3 mediated IL1β activation (Rajan et al., 2010). Nevertheless, Toll-like receptor signaling may result in augmented histone acetylation (Lauterbach et al., 2019), while histone deacetylases could act as regulators of inflammation and immunity (Shakespear et al., 2011). Consistent with the upregulation of *Rela* mRNA expression (Su et al., 2022), this study revealed a significant enhancement of both H3ace and H4ace peaking on the promoter of the *Rela* gene in the PFC of Poly I:C rats. However, even though there is also an elevated mRNA expression of *Nlrp3* (Su et al., 2022), ChIP-qPCR showed that there was not any significant enhancement of either H3ace or H4ace peaking on the promoter region of the *Nlrp3* gene in the PFC of the Poly I:C rats. These results suggest that prenatal Poly I:C exposure may help to hyperacetylate histone H4 and H3 on the promoter region of *Rela*, and thereby increase the expression of NF-κB, which will further modulate *Nlrp3* expression in the PFC.

HATs are the enzymes promoting histone acetylation events. Based on their cellular location and functions, they are grouped into two general classes (Roth et al., 2001); of them, A-type HATs were located in the nucleus (Roth et al., 2001). Although HATs could also directly bind on the specific promoter region which in turn regulates target gene expression (Roth et al., 2001; Sampley and Ozcan, 2012; Gupta et al., 2021), they modulate the transcriptional levels mainly through acetylating histone proteins in a space- and time-dependent manner. Individual type A HATs have been confirmed to be expressed in the brain and activated at specific times and locations to coordinate proper brain development (Sheikh, 2014). This study found that prenatal Poly I:C exposure caused an increased expression of two type A HATs (EP300 and CBP), but not the other three type A HATs (GRIN5, PCAF or TIP60), in the PFC. This is consistent with previous findings that the CBP/EP300-mediated acetylation of histone H3 and H4 are directly involved in epigenetic regulation of neurodevelopment and the pathologies of neuropsychiatric disorders (Valor et al., 2013; Li et al., 2017). Furthermore, significant positive correlations between *Rela* and gene encoding of HATs EP300 (*p300*) and CBP (*Crebbp*) were also observed in this study. Since the critical mechanism of A- HATs of EP300 and CBP in activating gene expression were through acetylating specific lysine residues in histones in the context of chromatin (Chan and La Thangue, 2001), these results suggested the enhancement of H3ace and H4ace on the promoter region of *Rela* in Poly I:C rats may be recruited by EP300 and CBP.

As opposed to HATs, HDACs are enzymes reversing chromatin acetylation by removing acetyl groups from histones (de Ruijter et al., 2003). Although prenatal Poly I:C induced the enhancement of H3ace and H4ace binding on the promoter region of target genes, no significant decrease of HDAC expression was found in this study, suggesting these HDACs were not involved in the histone acetylation events induced by prenatal Poly I:C exposure. On the other hand, it is consistent with the previous findings of improper activation of HDAC6 in a variety of inflammatory diseases (Ran and Zhou, 2019), that there was a significantly increased expression of HDAC6 in Poly I:C rats. Moreover, a significant positive correlation between the gene expression of HDAC6 and NLRP3, an important independent inflammation factor, was found in this study. These results suggested the involvement of HDAC6 in the pathogenesis of neuroinflammation induced by prenatal Poly I:C exposure. In fact, the underlying mechanisms of HDACs (including HDAC6) in promoting neuroinflammation are still not well understood (Yang et al., 2020; Dai et al., 2021). Beyond deacetylating the acetyl group on the C-terminal lysine of histones through the deacetylase domains of DAC1, HDAC6 may deacetylate cytoplasmic protein through the other deacetylase domains of DAC2 (Moses et al., 2020; Pulya et al., 2021). HDAC6 promoted neuroinflammation induced by the Human immunodeficiency virus-1 (HIV-1) transactivator of transcription (Tat) through activating the MAPK-NF-κB/AP1 signaling pathway or NADPH oxidase in astrocytes (Jo et al., 2018). A recent study also suggested that HDAC6 can act as a dyne in the adapter to mediate an aggresome-like mechanism for NLRP3 inflammasome activation through the transport of ubiquitinated pathological aggregates to the microtubule-organizing center (MTOC) for aggresome formation and autophagosomal degradation (Magupalli et al., 2020). Importantly, HDAC6 could act as a cofactor to be recruited to the promoters and directly activate the target gene expression (Palijan et al., 2009; Chen et al., 2013). In consideration of the positive correlation between the upregulation expression of *Nlrp3* and *Hdac6*, the enhanced *Hdac6* binding on the promoter region of *Nlrp3* in the ChIP-qPCR experiments suggested HDAC6 modulated *Nlrp3* expression through directly binding on the promoters, which in turn involved activation of the NF-κB /NLRP3 pathway induced by prenatal Poly I:C exposure.

**Figure 7:**
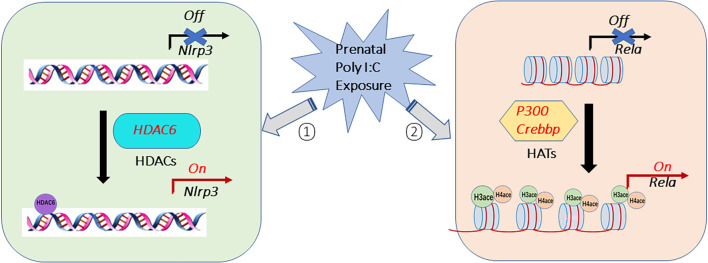
Potential epigenetic histone acetylation mechanisms for elevated neuroinflammation caused by prenatal Poly I:C exposure in the prefrontal cortex of female juvenile offspring rats. The activation of the NF-κB/NLRP3 inflammatory pathway induced by prenatal Poly I:C exposure may *via* ① activating histone acetyltransferases (HATs) of EP300 *(p300*) and CBP (*Crebbp*), which thus enhances histone H3 and H4 acetylation on the promoter region of *Rela*, a subunit of NF-κB, ② activating histone deacetylases of HDAC6 (*Hdac6*), which increases HDAC6-mediated NLRP3 transcriptional activation.

In summary, this study demonstrated that histone acetylation is involved in the prenatal Poly I:C-induced changes in the NF-kB/NLRP3 mediated inflammatory pathway in the PFC of juvenile female offspring (Figure 7). These changes are through modulating the enrichment of H3ace and H4ace in the promoter region of *Rela*. The results of this study also suggest that the hyperacetylation effect of histone H4 and H3 histone on *Rela* promoters in the PFC may be mediated by EP300 and CBP. This can be seen when considering the increased *Rela* expression and the higher acetylation of both histone H3 and H4 binding on the *Rela* promoter region in the ChIP-qPCR experiment, and the correlations between higher expression of *Rela* and genes of *p300* or *Crebbp* after the prenatal Poly I:C administration. Although there was no direct H3 and H4 histone acetylation at the promoter region of *Nlrp3*, the enhancement of HDAC6 peaking on the promoter region of *Nlrp3* and the correlation of higher expression of *Nlrp3* and *Hdac6* suggested HDAC6 may link to NLRP3 activation induced by prenatal Poly I:C exposure. These results suggest that epigenetic modulation of the NF-κB/NLRP3 inflammatory pathway contributes to neuroinflammation induced by prenatal Poly I:C exposure *via* enhancement of histone acetylation of H3ace and H4ace on *Rela* and HDAC6-mediated NLRP3 transcriptional activation (Figure 7), which may further lead to deficits in neurotransmissions and schizophrenia-like behaviors observed in offspring (Su et al., 2022). One of the limitations is that only female offspring rats have been examined in this study. Further studies are needed to investigate whether similar histone acetylation modifications are induced by prenatal Poly I:C exposure in male juvenile rats. The other limitation is that, due to the limitation of the small amount of the PFC tissue, this study has examined only histone H3 and H4 acetylation, but not on specific lysine residues. Therefore-further research should be extended to identify the acetylation of specific lysine residues in histones H3 and H4 for modulating neuroinflammation responses caused by prenatal Poly I:C exposure.

## Data Availability Statement

The raw data supporting the conclusions of this article will be made available by the authors, without undue reservation.

## Ethics Statement

The animal study was reviewed and approved by the Animal Ethics Committee, University of Wollongong, Australia.

## Author Contributions

YS, JL, CD, and WZ designed the experiments. YS, JL, and SC performed the experiments. YS and CD analyzed the data. YS prepared the initial draft of the manuscript. CD, YS, JL, SC, and WZ revised the manuscript. All authors commented on the final draft. All authors contributed to the article and approved the submitted version.

## Funding

This study was funded by the Australian National Health and Medical Research Council (NHMRC) Project Grant (APP 1104184) to CD and JL. JL was also supported by an NHMRC Early Career Fellowship Award (APP1125937). YS was supported by the Joint Funds for the Innovation of Science and Technology, Fujian Province (2020Y9144), and Natural Science Foundation of Fujian Province (2021J01412). The funding bodies had no further role in the study design, decision to publish or preparation of the manuscript.

## Footnotes

^1^ https://chip-atlas.org
