# Supplementary material for: Epigenetic histone acetylation modulating prenatal Poly I:C induced neuroinflammation in the prefrontal cortex of rats: a study in a maternal immune activation model
Source: Front Cell Neurosci. 2022 Nov 28;16:1037105. doi: 10.3389/fncel.2022.1037105 (PMC9742227; doi:10.3389/fncel.2022.1037105)
Supplement: Supplementary file 1 [file Data_Sheet_1.PDF]

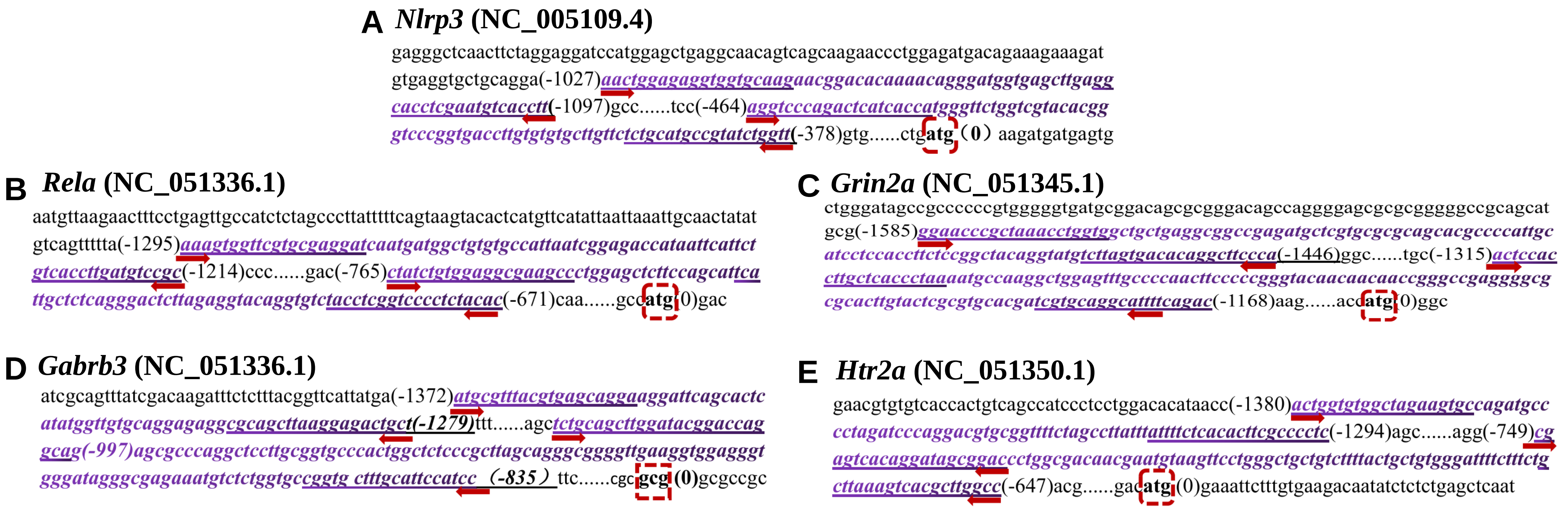

Supplementary Figure 1 Primer design for ChIP-qPCR. (A) *Nlrp3*, **(B)** *Rela*, **(C)** *Grin2a*, (**D)** *Gabrb*3, and **(E)** *Htr2a*. The red sequence blocked with dotted box is the position of initiation codon. The region of PCR amplification was presented with purple italics, and the extensional direction was indicated by a red arrow. The sequence marked with purple underlines are the PCR primers. The primers were self-designed through the Primer-BLAST in the National Center for Biotechnology Information (NCBI) (https://www.ncbi.nlm.nih.gov/tools/primer-blast/).

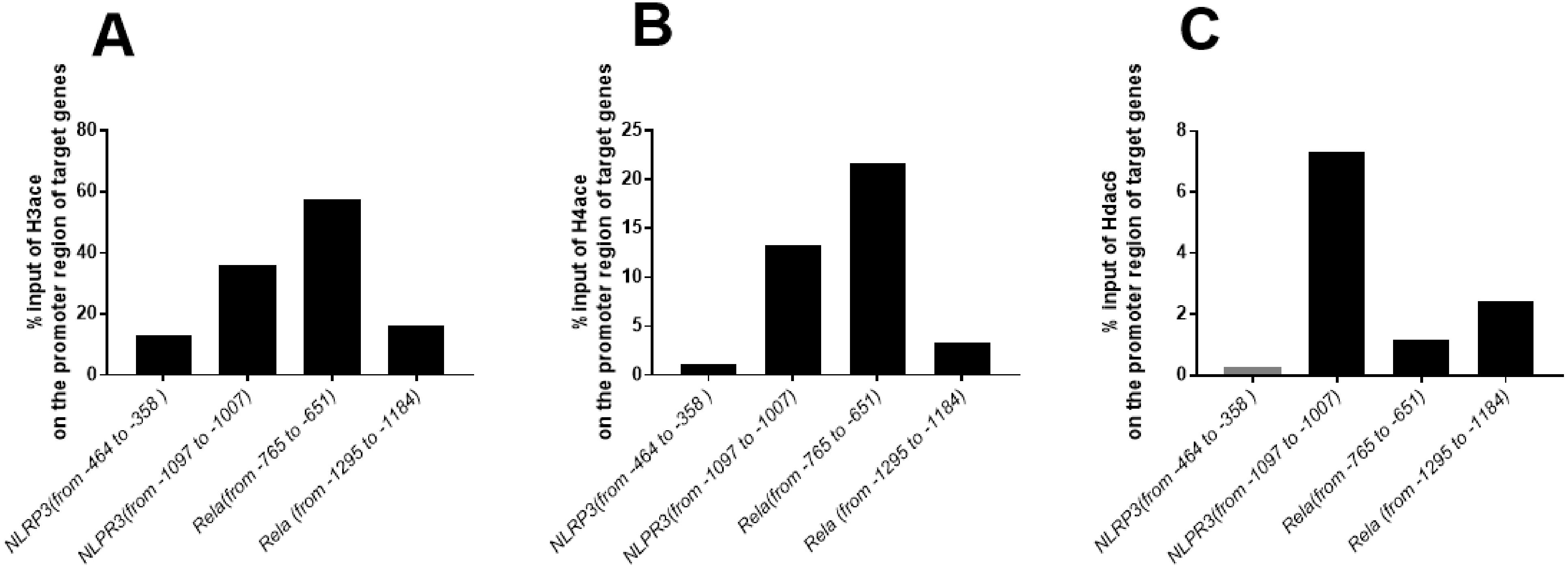

Supplementary Figure 2 Pre-experiment of ChIP-qPCR for *Nlrp3* and *Rela*. The condition of acetylated histones marking on the specified loci of promoter of target genes involved in neuroinflammation were evaluated by ChIP-qPCR using antibodies of **(A)** acetylated histone 3 (H3ace), **(B)** acetylated histone 4 (H4ace), and **(C)** Hdac6.

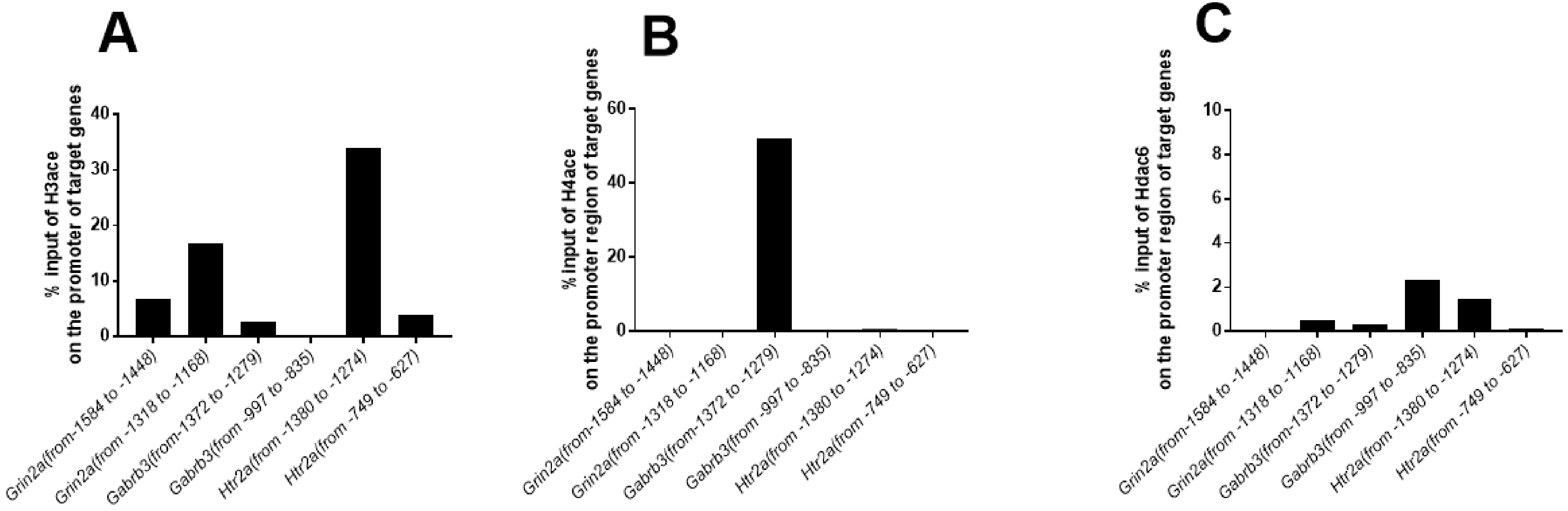

Supplementary Figure 3 Pre-experiment of ChIP-qPCR for neurotransmitter receptors (*Grin2a*, *Htr2a*, *Gabrb3*)**.** The condition of acetylated histones marking on the specified loci of promoter of target genes encoding neurotransmitter receptors was evaluated by ChIP-qPCR using antibodies of **(A)** acetylated histone 3 (H3ace), **(B)** acetylated histone 4 (H4ace) and **(C)** Hdac6.

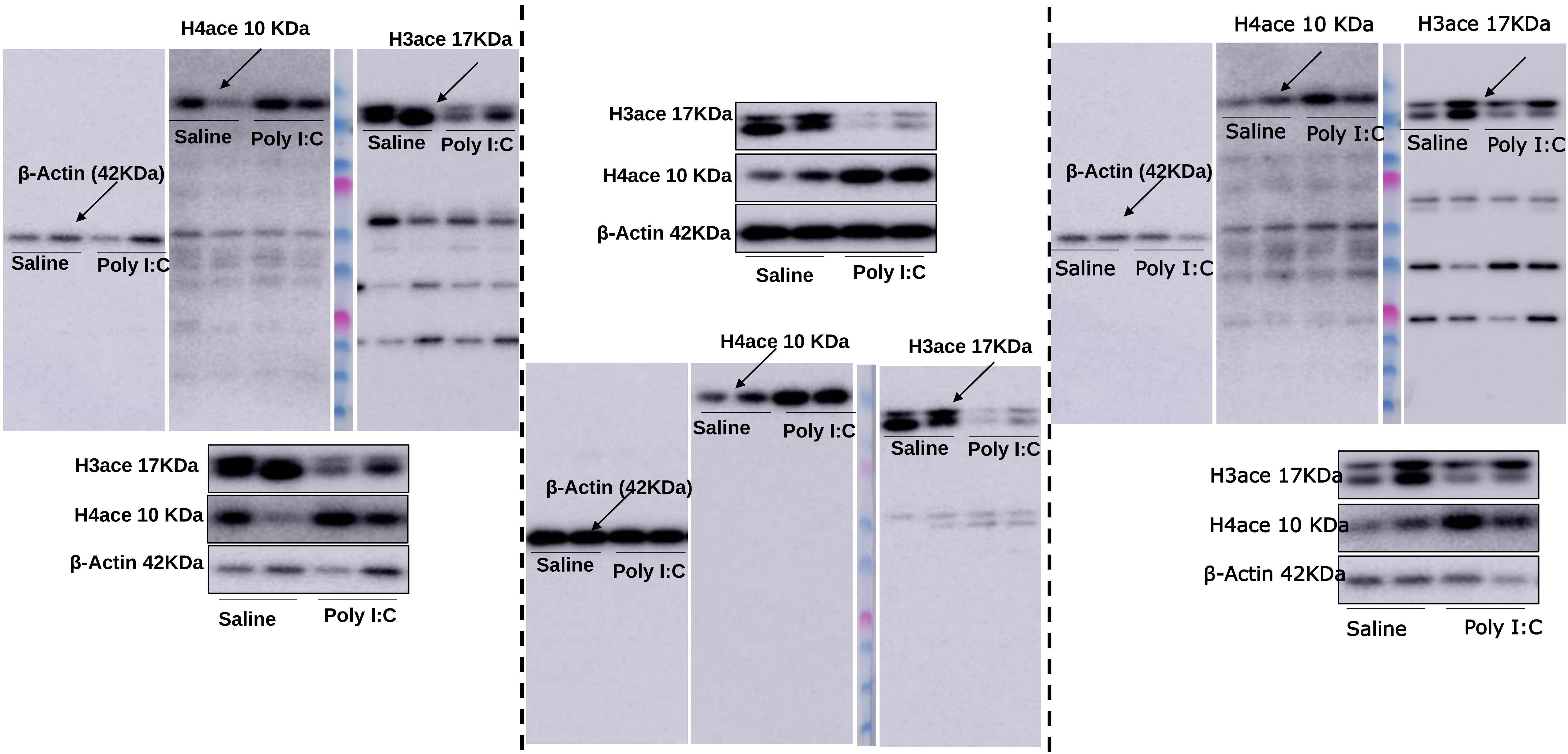

Supplementary Figure 4 Original western blot images of hepatic global H3ace and H4ace（n=6/group. The results were visualized by an Amersham GelImager 600. The molecular weights of H3ace, H4ace and Actin are 17kDa, 10kDa and 43kDa, respectively.
